# Supplementary material for: Polyketide synthase gene clusters in the ericoid mycorrhizal fungus Oidiodendron maius: genome-wide prediction, expression profiling, and functional characterization of knockout mutants
Source: Front Plant Sci. 2026 Sep 16;17:1924430. doi: 10.3389/fpls.2026.1924430 (PMC13623903; doi:10.3389/fpls.2026.1924430)
Supplement: Supplementary file 1 [file SupplementaryFile1.zip › Supplementary Materials/Supplementary Folder 1.zip!/Oidma1_AssemblyScaffolds_Repeatmasked/knownclusterblast/cluster17/jgi.p_Oidma1_120325_mibig_hits.html]

| MIBiG Protein | Description | MIBiG Cluster | MiBiG Product | % ID | % Coverage | BLAST Score | E-value |
| --- | --- | --- | --- | --- | --- | --- | --- |
| AEZ64549.1 | L-idonate\_5-dehydrogenase | BGC0001065 | Herboxidiene\_biosynthetic\_gene\_cluster | 31 | 97.150997151 | 131 | 1.9e-30 |
| BAD20754.1 | dehydrogenase | BGC0000702 | Kanamycin\_biosynthetic\_gene\_cluster | 30 | 95.4415954416 | 124 | 2.4e-28 |
| CAF60530.1 | GntP-like\_protein | BGC0000704 | Kanamycin\_biosynthetic\_gene\_cluster | 30 | 95.4415954416 | 124 | 2.4e-28 |
| CAF31584.1 | 3-amino-2,3-dideoxy-scyllo-inositol\_1-dehydrogenase | BGC0000705 | Kanamycin\_biosynthetic\_gene\_cluster | 30 | 95.4415954416 | 124 | 2.4e-28 |
| AAU04878.1 | polyketide\_synthase | BGC0000365 | Glycopeptidolipid\_biosynthetic\_gene\_cluster | 26 | 78.9173789174 | 74 | 2.8e-13 |
| CAF05649.1 | TubD\_protein | BGC0001053 | Tubulysin\_biosynthetic\_gene\_cluster | 23 | 80.056980057 | 57 | 2.7e-08 |
